# Pain assessment, cognitive and cortical changes with full mouth rehabilitation in a group of children

**DOI:** 10.1186/s12903-024-04356-w

**Published:** 2024-05-23

**Authors:** Nancy Mohamed Metwally, Osama Abd Allah Ragab, Mostafa Shaaban Husseiny Kandil, Lamis Ahmed Elghareb

**Affiliations:** 1 Pediatric Dentistry, Preventive Dentistry Department, Faculty of Dentistry, Oral Health, Tanta University, Tanta, Egypt; 2 Neurology Department, Faculty of Medicine, Tanta University, Tanta, Egypt; 3 Neuropsychiatry Department, Faculty of Medicine, Tanta University, Tanta, Egypt

**Keywords:** Cognitive functions, Cortical EEG changes, Full mouth rehabilitation, General anesthesia, Pain scale

## Abstract

**Background:**

A change in professionals’ perspectives on the value of general anesthesia (GA) for pediatric patients, including those with disabilities, medical conditions, severe oral issues, and challenging behaviors. Full-mouth rehabilitation under GA allows for the comprehensive treatment of all oral health problems in a single visit, without requiring the child’s active participation. Extensive dental problems are often associated with severe dental pain, which can impact cognitive function, including perception, attention, memory, reasoning, language, communication, and executive functions. Individuals experiencing pain tend to perform less optimally cognitively.

**Aim:**

This study aimed to investigate changes in cognition, brain function, and cortical alterations in children who underwent extensive dental rehabilitation under GA.

**Patients andMethods:**

Thirty uncooperative, healthy children aged 6–12 with extensive dental issues were enrolled. Pain levels were assessed using the FLACC and WBFPS scales before treatment, one week after, and three months later. Cognitive assessments, including the WCST, processing speed, digit span, and Trail Making Test, as well as EEG measurements, were also performed.

**Results:**

The results showed a significant improvement in pain levels reported by the children or their caregivers after the dental procedures, both at one week and three months. All cognitive measures, such as digit span, processing speed, and WCST performance, demonstrated substantial improvements after the treatment. The Trail Making Test also exhibited statistically significant variations before and after the dental procedures. Additionally, the MOCA test revealed a notable improvement in cognitive skills following the treatment. Furthermore, the EEG power ratio, an indicator of changes in the power balance within each frequency band, showed a statistically significant difference after the dental procedures.

**Conclusion:**

the findings of this study suggest that full-mouth rehabilitation under GA can lead to improved pain management, as well as enhanced cognitive and brain functions in children.

**Future perspectives:**

More clinical studies with a longer follow-up period and a different age range of children are required to investigate the connection between brain function and oral rehabilitation involving restorations or occlusion issues.

## Background

It has been clearly shown that poor oral care has a negative biological, psychological, and social impact on aesthetics and communication [1]. Children who have oral pain suffer terrible consequences, such as lack of sleep, stunted growth, behavioral issues, and poor academic performance [2, 3]. Dentalgia, or toothache (TA), is pain in the dental pulp and/or periodontal tissues caused by dental or non-dental diseases [4]. Since pain is essentially a personal experience, a variety of pain assessment tools (self-report and observational scales) have been utilized. The Faces Pain Scale (FPS) by Bieri et al. and the Numerical Rating Scale (NRS) by von Baeyer et al. are two widely used scales that demonstrate self-reporting of acute procedural, postoperative, or disease-related pain [5, 6]. Children with postoperative conditions can use observational scales to gauge their level of pain, such as FLACC (Face, Legs, Activity, Cry, and Consolability), especially for preverbal children and children who are unable to comprehend a self-report scale [7].

Cognitive functions are essential mental processes that enable perception, learning, memory, problem-solving, and decision-making. These processes must be coordinated and efficient to successfully navigate our complex world. Empirical evidence from animal studies has demonstrated that reduced masticatory activity due to pain and discomfort can lead to detrimental effects, including spatial memory impairment, diminished learning capacity, neuroendocrine dysregulation, and hippocampal degeneration. The hippocampus is a crucial brain region involved in memory formation and consolidation, among other functions. While the relationship between mastication and cognitive function has been explored in human populations, establishing causality has proven challenging due to the substantial heterogeneity in research cohorts and methodological approaches employed across studies [8]. Dental pain typically activates two central neural systems: the core pain-related network, predominantly organized by the primary somatosensory cortex (S1), and the cognitive-emotional network, primarily modulated by the prefrontal cortex (PFC). The PFC is associated with cognitive control, mainly the contextual biasing of attention to resolve conflicts and exert attentional control [9].

Studies have shown that individuals suffering from chronic pain, acute pain, or experimental pain have poor cognitive performance [10–15]. Therefore, receiving comprehensive dental care can potentially improve brain health. Full-mouth rehabilitation under general anesthesia (GA) is a treatment option for children who require substantial dental work, display severe situational anxiety, emotional or cognitive immaturity, or are in a medically fragile state [16]. It has various advantages, such as ensuring safety and comfort for children, saving dentist time and effort, efficiently completing lengthy procedures requiring multiple visits without wasting time and effort and distressing the child or parents, reducing the need for frequent multiple local anesthesia or conscious sedation visits in extensive restorations, offering a safer option, saving the family money, and reducing inconvenience [17]. The American Academy of Pediatric Dentistry (AAPD) asserts that GA can be used to treat a specific patient population that may not tolerate conventional dental therapy [18, 19]. Most dental GA candidates are young children who have early childhood caries (ECC), a common health issue, and children who exhibit excessive fear and anxiety during dental visits [20–23].

The relationship between oral health problems in children, including dental pain, and cognitive and brain function is not well understood and not clearly stated in previous studies. Therefore, this study aimed to assess pain and investigate its relation to cognitive and cortical alterations in children subjected to comprehensive dental rehabilitation under general anesthesia.

## Patients and methods

### Ethical considerations

The Research Ethics Committee of the Faculty of Dentistry at Tanta University granted approval for this study under the reference number (R-PED-11-22-11). The study’s objective was explained to the parents or guardians, and informed consent was obtained from the children’s legal guardians along with the assent of the children above the age of eight, in accordance with the ethical guidelines outlined in the Declaration of Helsinki and its subsequent revision.

### Sample size calculation

The sample size was calculated using G*Power version 3.1.9.2 [24]. Adopting a power of 80% (β = 0.20), and a level of significance of 5% (α error accepted = 0.05), to detect a standardized effect size (g) of 0.3, the minimum required sample size was found to be 25 patients [25]. After adjusting for a 10% dropout rate, the sample size was increased to 30 patients.

### Eligibility criteria

**Fig. 1:**
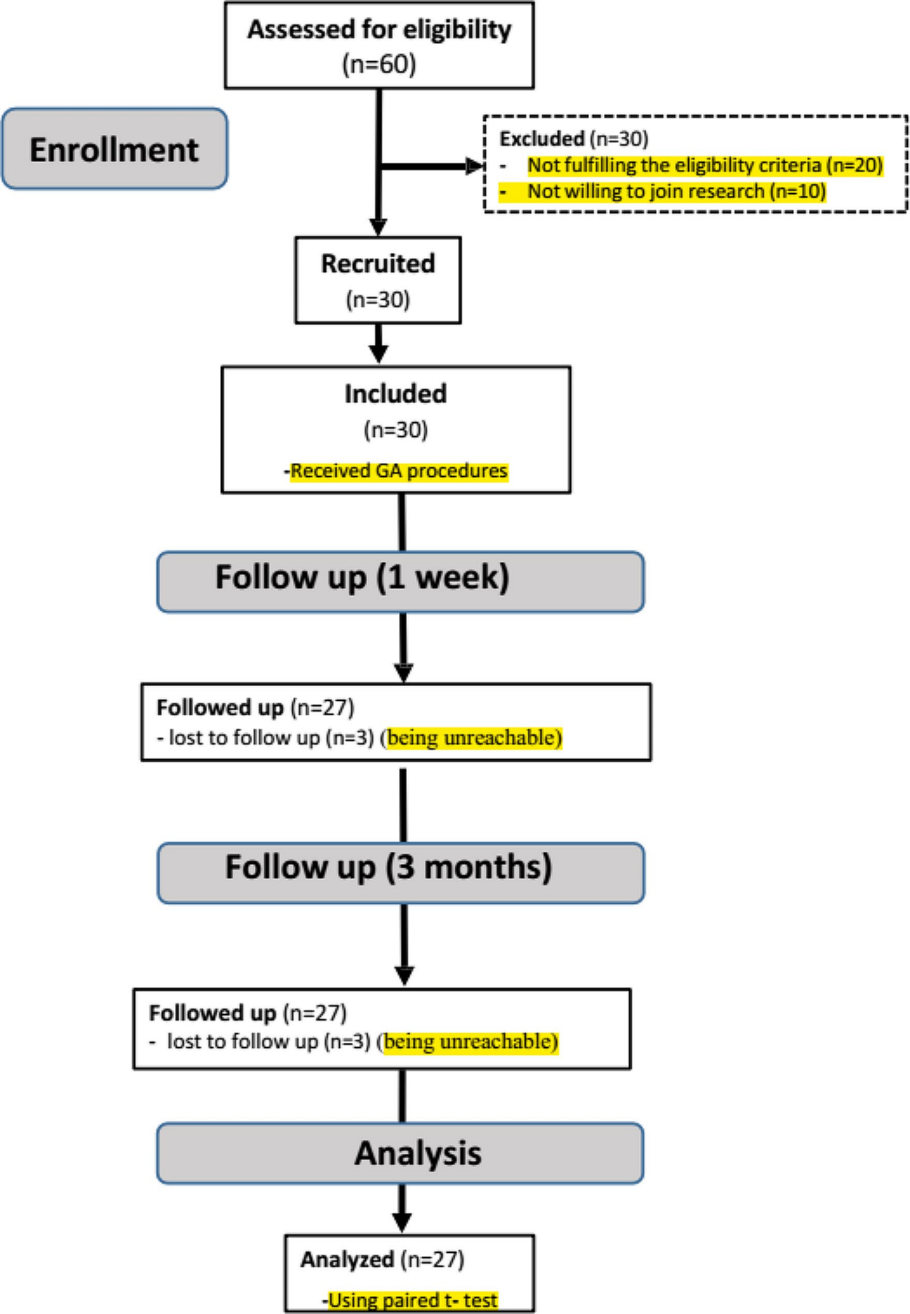
CONSORT Flow chart diagram of participants

This was a single-arm clinical trial. As shown in Fig. 1, sixty children were assessed for eligibility. Thirty patients were excluded: twenty did not meet the inclusion criteria, and ten declined to participate in the research. Thirty children were initially recruited for the study, but only 27 were followed up at 3 months. Three children lost to follow up. The study enrolled male and female patients, aged 6 to 12 years, with multiple oral problems. After one week, only 27 children (13 males and 14 females) were recruited from the outpatient’s pediatric dentistry clinic at the Faculty of Dentistry, Tanta University. Each selected child had severe situational anxiety, suggesting they were uncooperative. Both the ability for immobilization and effective communication approaches, or developing rapport with them, were unsuccessful. The supporting evidence affirmed the necessity of considering the use of GA.

#### Inclusion criteria

Healthy children (ASA I) with normal mental health and communication skills who exhibited uncooperative behaviour. Children who showed negative behaviour and were suffering from extensive dental procedures with severe dental pain.

#### Exclusion criteria

Patients or caregivers who were reluctant to participate. The presence of neurological disorders which includes seizures, neurodevelopmental disorders, and head injuries. Children with current or previous psychotic episodes or intellectual disabilities, e.g., down syndrome, autism, and mental disabilities.

### Procedures and methods of data collection [26]

The full-mouth rehabilitation procedure was performed in the pediatric anaesthesia unit of the public service centre, Faculty of Dentistry, Tanta University. Each child underwent a range of restorative procedures based on their age and case. These procedures included pulpotomies, pulpectomies for primary teeth, vital pulp and non-vital pulp therapies for permanent teeth, extractions for teeth that were nearly exfoliated, and space maintenance when necessary.

Children were evaluated for any complications after GA procedures. The most common postoperative consequences were dental pain, a slight sore throat, and nasal congestion that cleared up with decongestant drops. The complications were all minor and resolved in a few days.

**The participants were subjected to the following**:

### History taking

General and Neurological Examinations: to exclude any physical or neurological disorder. Pain evaluation scales, psychological scales, and EEG at baseline, and three months after completion of dental rehabilitation.

### Pain evaluation

**Fig. 2:**
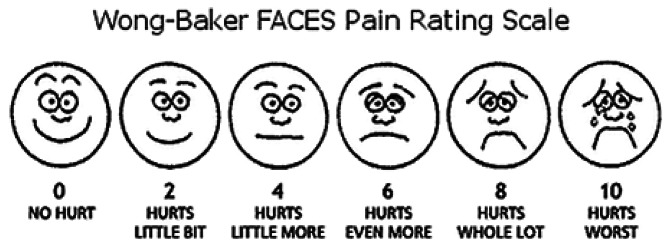
Wong-baker FACES pain rating scale [27]

Children’s pain levels were measured using the Wong-Baker Faces Pain Rating Scale (WBFPS). The children reported their pain levels using the WBFPS at three different time points: at baseline, just before the anesthetic treatments, and one week and three months after the dental procedures, respectively [27] (Fig. 2).

When the child was present in the clinic and at home, the accompanying parent was asked to rate their child’s pain tolerance and behavior using the Face, Legs, Activity, Cry, and Consolability Measure (FLACC), with a verified Arabic translation, at baseline, one week following the dental procedures, and three months after the procedures [28] (Table 1).

**Table 1:** FLACC scale for pain assessment from child behaviour (score 0–10) [28]

Behaviour	0	1	2
Face	No particular expression or smile	Occasional grimace or frown, withdrawn, disinterested	Frequent to constant quivering chin, clenched jaw
Legs	Normal position or relaxed	Uneasy, restless, tense	Kicking or legs drawn up
Activity	Lying quietly, normal position, moves easily	Squirming, shifting, back and forth, tense	Arched, rigid or jerking
Cry	No cry (awake or asleep)	Moans or whimpers; occasional complaint	Crying steadily, screams, sobs, frequent complaints
CONSOL ability	Content, relaxed	Reassured by touching, hugging, or being talked to, distractible	Difficult to console or comfort

### Cognitive function assessment

Stanford-Binet Intelligence Scale: The Arabic version of this scale was used to assess IQ for each subject [29]. The Arabic version of the Montreal Cognitive Assessment (MoCA) is a brief, one-page screening test used to identify potential cognitive impairment. It consists of 30 points, with scores of 26 or higher considered normal and scores below 26 indicating possible cognitive impairment [30].

Wisconsin Card Sorting Task (WCST): This task was used to test executive functions [31].

Trail-Making Test (TMT) or Trails A & B: Part A of this test measures processing speed, while TMT-B assesses set-shifting and cognitive flexibility (part of executive functioning). The Arabic version of this test was used [32].

Digit Span Subtest (DS): Participants had to repeat a mixed array of digits, first in the same order (forward span) and then in the opposite direction (backward span). The forward span is thought to measure basic attention, while the backward span is connected to working memory. The digit span is defined as the maximum number of successfully repeated digits before failing twice [33].

### Electroencephalogram (EEG)

Participants were instructed to close their eyes, refrain from thinking, and avoid moving or tensing their muscles while the EEG was being recorded. The Neurofax Nihon Kohden QP-110 AK system was used to record EEGs from scalp locations following the 10–20 international standard. Resting EEG was collected for five minutes from each patient to establish their baseline brain activity. The EEG was then digitized, and a fast Fourier transform (FFT) was performed.

The FFT is an algorithm that efficiently calculates the discrete Fourier transform of a sequence, or its inverse. It essentially transforms a signal from the time domain or space domain into the frequency domain, and vice versa, using Fourier analysis. The frequency spectrum was divided into 0.2 Hz bands, which were then grouped into the standard EEG frequency bands: beta (13.0–30 Hz), theta (4.0–7.9 Hz), alpha (8.0–12.9 Hz), and delta (1–3 Hz). This allowed for the calculation of EEG power. The FFT algorithm was used to analyze the frequency domain and determine the following for each sub-band: mean frequency (Hz), relative power (%), and absolute power density (µV²/Hz) [34].

**The following equations were utilized**:

- Mean band frequency in Hz: alpha at channel C3-P3 (at baseline and after 3 months of follow-up).
- Absolute alpha band power at channel C3-P3 (log ^x^) (This value is calculated as the area underlying the spectrum of the signal in the interval of frequency that defines alpha band and its measure unit is µV^2^/Hz).
- Relative alpha band power at channel C3-P3 (This value is calculated as the ratio between absolute power in and the total power and is normally expressed as a percentage).

Absolute power was log-transformed (log ^x^) to normalize the distribution of the data, while relative power variables were also transformed for the same purpose. EEG frequency (Hz) indices were normally distributed and therefore did not require transformation [35].

### Statistical analysis

Descriptive statistics were calculated using SPSS version 21 (IBM Corp, New York, USA), including number, frequency, mean, and standard deviations. The study variables were normally distributed, so t-tests were used for scale variables, and paired sample t-tests were used for before and after data comparisons.

## Results

Table 2 displays all demographic data, including the age and gender distributions of the enrolled children. Thirty children were included in the study; only 27 were followed up at 1 week and 3 months. Three children were unreachable for follow-up. For sex distribution, 48% of children were male, and 52% were female. The mean age was 9.2, ranging from 6 to 12 years old, with a mean ± SD of 9.23 ± 2.01.

**Table 2:** The study participants’ demographic distribution

Sex (*n* = 27)	Male			Female	
	N	%		N	%
	13	48		14	52
	Min		Max	Mean	SD
Age (*n* = 27)	6.00		12.00	9.23	2.01

### Pain evaluation

Before the GA dental procedures, all children suffered from severe pain, with scores above average (5/10). This was evident on both the WBFPS and FLACC scales reported by the children and parents, with mean ± SD of 7.96 ± 1.18 and 7.70 ± 0.79, respectively.

For the WBFPS, a paired t-test was applied to analyze differences in pain levels before and after the GA procedures. The pain scores significantly decreased one week after the GA procedures for the children. Most of the scores (mean = 2.36, *p* = 0.0) indicated minor to no pain. Three months after the GA treatments, there was a significant decrease in the pain scores (mean = 0.61), with almost no pain in all children, compared to the scores recorded before the GA procedures and one week after (*p* = 0.0). (Table 3)

The FLACC scale pain scores before and after the GA procedures were compared using the paired t-test. Children’s pain scores dramatically dropped one week after the GA procedure. Most of the scores (mean = 2.10, *p* = 0.0) indicated minor to no pain. Compared to the scores obtained one week after the GA treatments and before the procedures, the pain scores showed a significant decline (0.56) three months after the GA procedures, with almost no discomfort in any child (*p* = 0.0). (Table 4)

**Table 3:** Wong-Baker FACES Pain Rating Scale (WBFPS) comparisons prior to and following dental procedures (DP).

	Mean	SD	Paired t test	*P* value
Before DP (at baseline)	7.96	1.18	32.90	0.00*
After 1 week of DP	2.36	0.99		
Before DP (at baseline)	7.96	1.18	41.48	0.00*
After 3 months of DP	0.614	0.36		
After 1 week of DP	2.36	0.99	13.19	0.00*
After 3 months of DP	0.61	0.36		

^∗^Statistically significant at *p* < 0.05. **SD**: Standard deviation. DP: Dental Procedures

**Table 4:** FLACC scale comparison before and after the dental procedures (DP)

	Mean	SD	Paired t- test	*P* value
Before DP (at baseline)	7.70	0.79	34.29	0.00*
After 1 week of DP	2.10	0.84		
Before DP (at baseline)	7.70	0.79	45.83	0.00*
After 3 months of DP	0.56	0.43		
After 1 week of DP	2.10	0.84	10.81	0.00*
After 3 months of DP	0.56	0.43		

^∗^Statistically significant at *p* < 0.05. **SD**: Standard deviation. **DP**: Dental Procedures

### Cognitive function evaluation [36]

Regarding cognitive functions, when comparing the pre- and post-treatment data, there was a significant difference in all cognitive variables, including digit forward, digit backward, processing speed, WCST perseverative responses, and WCST failure to maintain set (Table 5). The Trail Making Test A and Trail Making Test B also showed statistically significant differences before and after the dental procedures (Table 6). The statistical analysis of the MoCA test results showed a significant increase in cognitive functions after the dental procedures (Table 7).

**Table 5:** Comparison of the WCST findings, processing speed, and (forward and backward) digits before and after the dental procedures (DP).

			Paired t- test	*P* value
Digit forward before DP	Mean	3.75	-3.80	0.001*
	SD	0.58		
Digit forward after DP	Mean	4.32		
	SD	0.42		
Digit backword before DP	Mean	2.43	-5.49	0.001*
	SD	0.74		
Digit backword after DP	Mean	3.42		
	SD	0.71		
Processing speed before DP	Mean	81.53	-4.23	0.001*
	SD	6.63		
Processing speed after DP	Mean	89.39		
	SD	8.47		
WCST(PR) before DP	Mean	24.17	2.065	0.048*
	SD	4.58		
WCST (PR) after DP	Mean	18.23		
	SD	15.34		
WCST (FMS) before DP	Mean	7.33	8.564	0.001*
	SD	2.11		
WCST(FMS) after DP	Mean	3.4		
	SD	0.79		

^∗^Statistically significant at *p* < 0.05. **SD**: Standard deviation. **DP**: Dental Procedures

**Table 6:** Comparison of trial make tests (TMT) results before and after the dental procedures (DP).

	Mean	SD	Paired t- test	*P* value
TMT A (Time) before DP	54.04	17.34	2.90	0.002*
TMT A (Time) after DP	40.08	17.41		
TMT A (Error) before DP	5.20	1.34	2.84	0.008*
TMT A (Error) after DP	4.28	1.09		
TMTB (Time) before DP	126.6	25.26	7.87	0.001*
TMTB (Time) after DP	87.72	12.63		
TMTB (Error) before DP	8.56	1.40	3.28	0.003*
TMTB (Error) after DP	7.37	1.13		

^*^Statistically significant at *p* < 0.05. **SD**: Standard deviation. **DP**: Dental Procedures

**Table 7:** Comparison of Montreal Cognitive Assessment (MoCA) results before and after the dental procedures (DP).

	Mean	SD	Paired t- test	*P* value
MoCA before DP	24.06	1.28	-10.24	0.001*
MoCA after DP	27.60	1.56		

^*^Statistically significant at *p* < 0.05. **SD**: Standard deviation. **DP**: Dental Procedures

Power ratios are a measure of EEG power that show variations in the power balance within each frequency band. The current results found a significant difference between the baseline values of alpha relative power and absolute alpha power before and after the dental procedures (Table 8).

**Table 8:** Comparison of EEG indices before and after the dental procedures (DP).

	Mean	SD	Paired t -test	*P* value
Alpha range before DP (at baseline)	9.90	1.18	1.30	0.23
Alpha range after DP	9.53	1.13		
Absolute alpha power before DP (at baseline)	1.95	0.56	-7.24	0.001*
Absolute alpha power after DP	2.82	0.24		
Relative alpha power before DP (at baseline)	55.90	20.92	-4.24	0.001*
Relative alpha power after DP	75.46	20.49		

^*^Statistically significant at *p* < 0.05. **SD**: Standard deviation. **DP**: Dental Procedures

## Discussion

Unfortunately, most of the research that has been published has investigated the quality of life, cognitive function, and brain correlates of older adults following oral rehabilitation [37–60]. Even yet, most pediatric dental research has focused on pain assessment during dental rehabilitation or oral health-related quality of life [41–43]. Based on this, the current study aimed to investigate the relationships between oral rehabilitation for children and its impact on mental and cognitive functions.

Most children can receive dental care in a conventional setting, but some patients do not respond well to typical behavior modification techniques [44, 45]. Children who undergo dental care under GA benefit from instant pain relief and one-visit full-mouth rehabilitation. Crucial dental restorations can be completed in a single session due to its improved efficiency [45, 46].

Children’s self-report is the gold standard for assessing pain, as it is a subjective experience rather than a clinical diagnosis [47]. In our study, pain was measured using a modified VAS that employed facial expressions. The Wong-Baker scale, which has also been effectively used with children, and this modified version of the VAS have a good association, according to previous research. However, the Wong-Baker scale has been linked to overestimation of pain because apprehensive children who are not in pain might not choose a happy face on the scale [48]. Furthermore, the unique characteristics of each patient, their family history, and other relevant data for pain assessment make clinical decisions more difficult [49]. When parents’ views and judgments are included in the pain assessment, it may be more accurate than one that only considers the patient’s experience and the clinician’s observations because parents are aware of their child’s typical pain reactions and appreciate contextual and systemic information [50]. The fundamental behavioral categories in the FLACC pain assessment tool have been consistently linked to pain in cognitively intact individuals [51, 52].

In the present study, there was no difference between parents’ and their children’s ratings. All of the children experienced severe pain prior to receiving dental procedures. This was evident on the WBFPS and FLACC scales. This tool has been evaluated against various criteria in previous studies of children during their early and later surgical recovery phases [51–53]. While some studies have shown variances, others have found that the parent and child’s reported pain levels coincide [54–56]. In this study, pain was evaluated one week after the completion of dental rehabilitation and three months of follow-up. Dental pain was evaluated at one week, as the most frequently and persistently reported postoperative morbidity indicators and symptoms in children receiving GA treatment were dental discomfort and low appetite, as reported by Rajab and associates [57]. This pain lasts until the seventh day following GA procedures [58]. Pain was evaluated after three months to ensure the resolution of pain and its relation to cognitive functions.

The pain has significantly decreased after one week of evaluation. And there was another considerable decrease in the pain score after three months of evaluation using both scales. This matches the findings of Alohali et al. [58], who concluded that after a week, 99% of families were satisfied with the care their children had received, and 11% of families reported post-operative morbidity. These improvements also coincide with previous research on parents’ perceptions of their children’s quality of life, which revealed that, following dental treatment under GA, pain reduction was the most important factor, followed by improvements in sleeping and feeding patterns [59, 60]. Additionally, a study by Versloot and colleagues [61] evaluated whether the Dental Discomfort Questionnaire pain-related behaviors were persistent and conducted a follow-up study to assess how dental therapy affects pre-schoolers’ pain-related behaviours. They concluded that children who receive dental care have lower pain-related behaviours, which improves their quality of life.

Our results revealed significant differences in all cognitive variables pre- and post-procedure, including digit forward, digit backward, processing speed, WCST perseverative responses, WCST failure to maintain set, and the Trail Making Test. Statistical analysis of the MOCA also showed a significant improvement in cognitive functions after the dental procedures.

Despite studying different populations, Gu and colleagues [62] reported similar findings in their work. They found that poor periodontal status was strongly associated with worse global cognitive performance, especially in short-term memory and executive function, in the aging population. Their study also demonstrated an association between oral health and global cognition.

A previous systematic review found the most consistent associations between oral health and the cognitive domains of learning/memory, complex attention, and executive function. Other studies found oral health predicted performance in these domains even after accounting for confounding factors [63].

Electroencephalography (EEG) directly measures cortical activity involved in cognition and emotion. Therefore, EEG power spectral analysis is a valuable objective index of psychological state. In recent years, alpha, beta, and theta band powers have been used to evaluate psychological state. Alpha waves are prominent during resting wakefulness with eyes closed, while beta waves increase during concentration and mental effort [64]. While clinical assessments remain the primary evaluation method for most cognitive functions, EEG serves a complementary role by providing valuable insights into cortical information processing and neurophysiological processes underlying cognitive domains. EEG power, representing the synchronous discharge of neurons, has been proposed as a potential measure reflecting the capacity or performance of cortical information processing, with some studies reporting positive correlations between higher intelligence quotient (IQ) and increased absolute alpha and beta band power, as well as decreased delta and theta band power. A continuum of relationships between EEG and cognitive function has been reported, with significant correlations observed between EEG measures and neuropsychological performance, demonstrating the predictive validity of EEG in assessing cognitive abilities [65].

The current study showed a significant difference in the mean log-transformed alpha band power and alpha band relative power at baseline before performing any dental procedure compared to after the dental procedure.

These findings are consistent with Saikia et al. [66], who analyzed the influence of fixed dental prostheses on brain function. Cognitive function was assessed using a mental state questionnaire, and EEG alpha wave power spectral density analysis was conducted pre-treatment and post-treatment. They demonstrated improved brain function in partially edentulous patients after rehabilitation. Changes in EEG can be explained by reduced imbalance in trigeminal proprioceptive signalling, thereby improving performance on complex sensorimotor tasks and increasing prefrontal cortex activation [63]. Additionally, Silva Ulloa and colleagues [67] suggested that structural changes in the oral and masticatory system may trigger alterations in brain function. Consequently, it can be hypothesized that dental treatments targeting these structural issues could potentially have a positive impact on mental health by addressing the underlying neurological changes.

To the best of our knowledge, this is the first research that assesses dental pain and its relation to brain and cognitive functions in children after oral rehabilitation. The study’s findings indicate that after dental procedures, children’s dental pain and brain cognitive functions significantly improved. Nevertheless, to draw more definitive conclusions, further clinical research examining the relationship between brain functioning and oral rehabilitation involving restorations or occlusion problems, with a longer follow-up period and a different age group of children, is needed. While the current study has some limitations, such as a small sample size, a single group without a comparison group, and short follow-up periods, its findings can nevertheless be applied to other studies.

## Conclusion

The research findings indicate that full-mouth rehabilitation under GA improved pain scales as reported by both the children and their parents/caregivers. It also improved all cognitive and brain functions, including digit forward, digit backward, processing speed, WCST perseverative responses, WCST failure to maintain set, Trail Making Test, and EEG indices, when comparing the pre- and post-procedure values in the children. These results demonstrate the importance of full-mouth rehabilitation under GA to enhance cognitive and brain abilities in children.

## Data Availability

On reasonable request, the datasets utilized and/or analyzed during the present study are accessible from the corresponding author.
